# The association of birth weight and current BMI on the risk of hypertension: the Tohoku medical megabank community-based cohort study

**DOI:** 10.1038/s41440-024-01827-z

**Published:** 2024-08-08

**Authors:** Hiromi Himuro, Mana Kogure, Naoki Nakaya, Tomohiro Nakamura, Rieko Hatanaka, Ippei Chiba, Kumi Nakaya, Naho Tsuchiya, Takumi Hirata, Masatsugu Orui, Tomoko Kobayashi, Eiichi N. Kodama, Yohei Hamanaka, Akira Uruno, Nobuo Fuse, Satoshi Nagaie, Soichi Ogishima, Mami Ishikuro, Taku Obara, Yoko Izumi, Masatoshi Saito, Shinichi Kuriyama, Atsushi Hozawa, Junichi Sugawara

**Affiliations:** 1 Graduate School of Medicine, Tohoku University, Sendai, Japan; 2 Tohoku Medical Megabank Organization, Tohoku University, Sendai, Japan; 3 Faculty of Data Science, Kyoto Women’s University, Kyoto, Japan; 4 Yamato Home Medical Care Clinic Kurihara, Kurihara, Japan; 5 Institute for Clinical and Translational Science, Nara Medical University Hospital, Nara, Japan; 6 Suzuki Memorial Hospital, 3-5-5 Satonomori, Iwanuma, Miyagi Japan

**Keywords:** Birth weight, Body mass index, Hypertension

## Abstract

This study aimed to investigate the association of combination of birth weight and current body mass index (BMI) with the risk of hypertension in adulthood. This cross-sectional study used data from the Tohoku Medical Megabank Community-based Cohort Study conducted in Japan. A total of 10,688 subjects aged ≥20 years were eligible. We calculated the least square (LS) means of systolic blood pressure (SBP) and trend tests were performed to evaluate the linear relationships between birth weight categories and SBP. We also used a multivariate logistic regression analysis to assess the risk of hypertension associated with the combination of birth weight and current BMI. There was a statistically inverse association between birth weight and SBP in the 20–64 age group, but no significant association in the ≥65 age group. Low birth weight (LBW) with normal BMI group had a higher risk of hypertension than the normal or high birth weight groups with normal BMI. Furthermore, the group with LBW and BMI ≥25.0 kg/m^2^ was the highest risk for hypertension (adjusted odds ratio: 2.73; 95% CI, 2.04–3.65) compared to the reference group (birth weight 2500–3499 g and BMI 18.5–24.9 kg/m^2^). There was a significant association between LBW and subsequent risk of hypertension. In addition, participants with lower birth weights had a higher risk of hypertension than those with higher birth weights. However, even in participants with a lower birth weight, the risk of hypertension could be reduced when they maintained an optimal BMI.

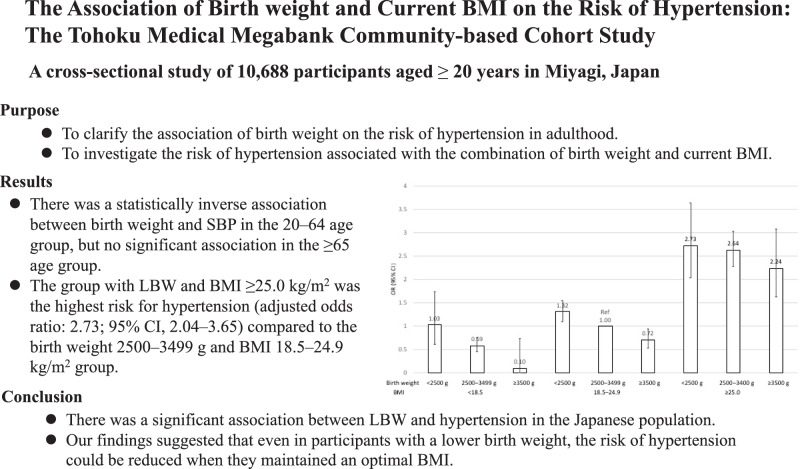

## Introduction

Hypertension is the leading risk factor for death and disability worldwide, and a major factor responsible for heart disease, stroke, and kidney failure. In 2019, the global prevalence of hypertension in adults aged 30–70 years was 34% in men and 32% in women; it has been on an increasing trend attributed to population growth and aging [1]. Hypertension is caused by multiple genetic and environmental factors such as obesity, excessive salt intake and alcohol consumption, smoking, and lack of physical activity [2]. Recently, birth weight has been suggested to be associated with hypertension [3–5].

Low birth weight (LBW) is defined as birth weight <2500 g by the World Health Organization (WHO) [6]. Globally, LBW accounted for 20.6 million of all births (i.e., 15.5%) by 2020 [7]. Many developing countries have shown a high prevalence of LBW, particularly South-Central Asia (27.1%) and Africa (14.3%) [7]. On the other hand, the prevalence of LBW is lower in the developed countries of North America (7.7%) and Europe (6.4%) [7]. However, in Japan, the prevalence rate of LBW infants has been increasing since the 1980s (5.2% in 1980, 9.2% in 2020) [8]. Increasing maternal age, multiple pregnancies, obstetric complications, underweight status in Japanese women, and low gestational weight gain may contribute to LBW [9, 10].

The Developmental Origins of Health and Disease hypothesis suggests that environmental status during the fetal and infant periods affects the onset of diseases such as hypertension, diabetes mellitus, and dyslipidemia [3]. Based on this hypothesis, epidemiological studies in a variety of age groups from childhood to old age have reported that birth weight is inversely associated with the risk of hypertension [4, 5]. However, most of these studies were conducted in limited age groups. No reports have covered a wide range of age groups, and it is unclear whether the association between birth weight and hypertension in later life varies with age.

In addition, it has been reported that subjects who were born small and had obesity later in life had a particularly high risk of hypertension [11, 12]. However, it was not known whether participants with lower birth weight had no excess risk of hypertension if they maintained optimal BMI revels.

Additionally, it is unclear whether the negative effects of birth weight on blood pressure (BP) can be reduced by preventing obesity. Therefore, an investigation to assess the association between birth weight and adult body mass index (BMI) on the risk of hypertension is needed.

This study aimed to clarify the association of birth weight on the risk of hypertension in adulthood. We investigated the association between birth weight and current BP by age group and the risk of hypertension associated with the combination of birth weight and current BMI using large cohort data covering a wide range of age groups.

Point of view

- Clinical relevance
  There was a significant association between LBW and subsequent risk of hypertension. However, even in those with a lower birth weight, the risk of hypertension could be reduced when they maintained an optimal BMI.
- Future direction
  We were unable to distinguish between premature and growth-restricted infants in this study. A further study is needed to investigate these differences.
- Consideration for the Asian population
  In Asia, there is a high proportion of people with low birth weight and low BMI. We believe that this raises important issue regarding the problem of hypertension in Asia. Replication studies in other populations are expected.

## Methods

### Study population

The Tohoku Medical Megabank (TMM) project was conducted by the Tohoku Medical Megabank Organization, Tohoku University, Miyagi Prefecture, northern Japan [13–15]. It was launched to realize creative reconstruction and solve medical problems in the aftermath of the Great East Japan Earthquake of 2011. The Tohoku Medical Megabank Community-based Cohort Study (TMM CommCohort Study) was a population-based prospective cohort study in Miyagi or Iwate Prefecture, northeastern Japan [14].

This cross-sectional study used data from the TMM cohort study. In this study, only participants recruited in the Miyagi Prefecture were included. Participants were recruited for this study between May 2013 and March 2016. Type 1 survey (40,433 participants) was conducted at a municipal-specific health checkup site. Here, information was documented based on blood and urine samples, questionnaires, and municipal health checkups. Type 1 additional survey (664 participants) was conducted on different dates from those of the municipal health checkups. Type 2 survey (13,855 participants) was conducted at a community support center. Here, in addition to the information pattern collected in the Type 1 survey, several physiological measurements were collected. In the Type 1 survey, 3833 participants underwent detailed physical measurements at a community support center. In total, 17,688 participants were included in this study. All participants provided written informed consent to participate in this study, as approved by the Institutional Review Board of Tohoku Medical Megabank Organization (approval number: 2012-4-617, 2023-4-044).

To be included in the analysis, the participants were required to undergo several physiological measurements. In addition to the participants of the Type 2 survey (13,855 participants), those who underwent physiological measurements at the community support center after the Type 1 survey (3833 participants) were included. All participants completed a self-administered questionnaire including medical history, family history, and lifestyle factors (smoking, alcohol consumption, and physical activity). Additionally, detailed physiological measurements (height, weight, BP, carotid ultrasound imaging, calcaneal ultrasound, and bone mineral density) and blood tests (non-fasting state) were conducted at the community support center.

**Fig. 1:**
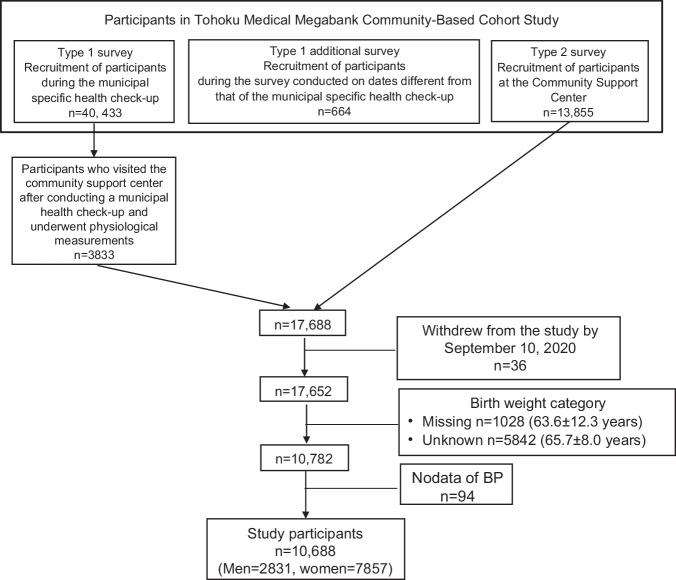
Flowchart of study participants. BP blood pressure

A total of 17,688 participants were included in this study. Figure 1 shows a flowchart illustrating the participant selection. We excluded the following: withdrew from the study by September 10, 2020 (*n* = 36), birth weight category “no reply” (*n* = 1028) or responded “unknown” (*n* = 5842), no data of BP (*n* = 94). Ultimately, 10,688 individuals (2831 men and 7857 women) were included in the analysis.

### Assessment of birth weight

Data on birth weight was based on recall as reported by the participants. The participants were asked to identify their birth weight from the following categories of responses: <1500 g, 1500–1999 g, 2000–2499 g, 2500–2999 g, 3000–3499 g, 3500–3999 g, ≥4000 g, and unknown. From a total of 17,688 participants, 10,688 participants (60.4%) provided information on their birth weight.

### BP measurement

BP was measured at a community support center after resting in sitting position for ≥2 min. Measurements were taken twice in the upper right arm using a digital automatic BP monitor (HEM-9000AI; Omron Healthcare Co., Ltd., Kyoto, Japan). The average values of the two recorded measurements were used in the analysis.

### Data and definitions

We included potential confounding factors, such as age, sex, BMI, smoking and alcohol status, treatment for hypertension, and blood test data (total cholesterol and HbA1c). Age was determined during the community support center visit. Based on height and weight measured at the community support center, BMI was calculated as the weight divided by the height squared (kg/m^2^), and obesity was defined as a BMI ≥25.0 kg/m^2^. The information on treatment for hypertension was obtained by a self-reported questionnaire. The participants chose one opinion from the following responses: (a) under treatment for hypertension, (b) discontinued hypertension treatment, (c) undertaking lifestyle modification without medication, (d) under observation without medication, or (e) never been diagnosed with hypertension. Individuals who answered (a) were classified as ‘under treatment’ while those who responded with (b)–(e) were classified as ‘without treatment’. Hypertension was defined as systolic SBP ≥140 mmHg and/or diastolic blood pressure (DBP) ≥90 mmHg and/or under treatment. Participants who reported on undergoing treatment for hypertension in the questionnaire were defined as having hypertension. Smoking status was classified into three categories: never smoked, ex-smoker, and current smoker. Alcohol status was classified into four categories: never-drinker, ex-drinker, current-drinker <23g/day, and current-drinker ≥23g/day. We determined the cutoff value of alcohol consumption as 23 g because it is the traditional Japanese unit of *sake*.

Birth weight was categorized into the following three groups: <2500 g, 2500–3499 g, and ≥3500 g. We defined birth weight <2500g as the LBW group and ≥3500 g as the high birth weight group.

Age was categorized into the following three groups: 20–39 years, 40–64 years, and ≥65 years. We defined 20–39 years as younger-aged, 40–64 years as middle-aged, and ≥65 years as older-aged. BMI was categorized into three groups: <18.5 kg/m^2^, 18.5–24.9 kg/m^2^, and ≥25.0 kg/m^2^.

### Statistical analysis

Data are presented as mean (standard deviation) for continuous variables and as frequency (percentage) for categorical variables. To compare the baseline characteristics of the birth weight category, we used one-way analysis of variance for continuous variables and Chi-square tests for categorical variables (prevalence of hypertension, diabetes, dyslipidemia, obesity, and smoking and drinking status).

We calculated the least square (LS) means of SBP, and trend tests were performed to evaluate the linear relationship between birth weight categories and SBP. We conducted a stratified analysis based on age or sex to examine the effects of these factors.

Additionally, we investigated the relationship between the presence of hypertension and the combination of birth weight and BMI to determine the effect of current BMI on the association between birth weight and hypertension. Participants were classified into nine groups based on a combination of their birth weight and BMI categories; multivariate logistic regression analysis was carried out to calculate odds ratios (ORs) and 95% confidence intervals (95% CI) for hypertension with a combination of birth weight and BMI. We used the group with birth weight 2500–3499 g and BMI 18.5–24.9 kg/m^2^ as the reference group. These analyses were conducted using the following models: crude, adjusted for age and sex, and multivariate model (adjusted for age, sex, BMI, total cholesterol, HbA1c, smoking status, and alcohol status). BMI was excluded from the logistic analysis. In the interaction term analysis, we included a cross-product term for the birth weight and BMI categories.

All statistical analysis were performed using the SAS software, version 9.4 for Windows (SAS Institute Inc., Cary, NC, USA). We considered p < 0.05 as statistically significant.

## Results

### Baseline characteristics

Table 1 shows the characteristics of the study participants. The mean age (±standard deviation) was 53.4 (±13.3) years. Study participants were significantly younger than those without information on birth weight. Age and prevalence of hypertension, diabetes, and dyslipidemia were significantly higher in the LBW group than in the high birth weight group. Birth weight category was positively associated with current BMI (*p* = 0.0026), obesity (*p* <0.0001), current smoking (*p* = 0.0001), and current drinking (*p* <0.0001).

**Table 1:** Baseline characteristics of participants according to birth weight category

		Birth weight category			*p* value
		<2500 g	2500–3499 g	≥3500 g	
Number	10,688	1576 (14.7)	8224 (77.0)	888 (8.3)	
Age (years), mean (SD)	53.4 (13.3)	57.6 (12.8)	53.1 (13.2)	48.5 (13.3)	<0.0001
20–39	1892 (17.7)	181 (9.6)	1475 (77.9)	236 (12.5)	0.6590
40–64	6297 (58.9)	835 (13.3)	4932 (78.3)	530 (8.4)	<0.0001
≥65	2499 (23.4)	560 (22.4)	1817 (72.7)	122 (4.9)	0.8981
Sex, number (%)					0.0402
Men	2831 (26.5)	454 (28.8)	2131 (25.9)	246 (27.7)	
Women	7857 (73.5)	1122 (71.2)	6093 (74.1)	642 (72.3)	
BMI, mean (SD)	22.7 (3.5)	22.7 (3.4)	22.6 (3.5)	23.3 (3.8)	0.0026
Obesity, number (%)	2459 (23.0)	353 (22.4)	1844 (22.4)	262 (29.5)	<0.0001
SBP, mean (SD)	125.8 (17.7)	129.1 (18.4)	125.6 (17.6)	121.8 (17.1)	<0.0001
DBP, mean (SD)	77.4 (11.0)	77.9 (11.1)	77.4 (11.0)	76.1 (10.8)	0.0003
Hypertension, number (%)	3643 (34.0)	683 (43.3)	2742 (33.3)	218 (24.6)	<0.0001
Treatment for hypertension, number (%)	1760 (17.9)	378 (26.9)	1288 (17.0)	94 (11.2)	<0.0001
HbA1c, mean (SD)	5.4 (0.5)	5.5 (0.5)	5.4 (0.4)	5.3 (0.4)	<0.0001
Total cholesterol, mean (SD)	207.7 (35.8)	207.8 (35.6)	208.2 (35.7)	203.4 (36.7)	0.0288
Smoking status, number (%)					<0.0001
Never smoked	6766 (63.3)	1045 (66.3)	5213 (63.4)	508 (57.2)	
Ex-smoker	2542 (23.8)	341 (21.6)	1968 (23.9)	233 (26.2)	
Current smoker	1338 (12.5)	176 (11.2)	1019 (12.4)	143 (16.1)	
Unknown	42 (0.4)	14 (0.9)	24 (0.3)	4 (0.5)	
Alcohol status, number (%)					0.0001
Never-drinker	3806 (35.6)	575 (36.5)	2963 (36.0)	268 (30.2)	
Ex-drinker	244 (2.3)	50 (3.2)	174 (2.1)	20 (2.3)	
Current <23 g/day	4118 (38.5)	565 (35.9)	3194 (38.8)	359 (40.4)	
Current ≥23 g/day	1870 (17.5)	286 (18.1)	1395 (17.0)	189 (21.3)	
Unknown	650 (6.1)	100 (6.3)	498 (6.1)	52 (5.8)	

Obesity was defined as a BMI ≥25.0 kg/m^2^. Hypertension was defined as SBP ≥140 mmHg and/or DBP ≥90 mmHg and/or under treatment

*BMI* body mass index, *DBP* diastolic blood pressure, *SBP* systolic blood pressure, *SD* standard deviation

### Birth weight and LS mean of SBP

Table 2 shows the relationship between birth weight and the LS mean of SBP by age category. A statistically significant inverse association was observed between birth weight and SBP. We found that SBP was the highest in the LBW group (10,688 participants). This trend did not change when adjusted for age, sex, and other multivariate models. When stratified according to age group, a similar association was observed in the middle-aged group as seen in the analysis with total participants. In the younger age group, although a significant association between birth weight and SBP was also observed in multivariate model. However, there was no significant association between birth weight and SBP in the older age groups.

**Table 2:** Birth weight and least square means of systolic blood pressure

Total													
		Total *n* = 10,688			20–39 years *n* = 1892			40–64 years *n* = 6297			≥65 years *n* = 2499		
		<2500 g	2500–3499 g	≥3500 g	<2500 g	2500–3499 g	≥3500 g	<2500 g	2500–3499 g	≥3500 g	<2500 g	2500–3499 g	≥3500 g
		*n* = 1576	*n* = 8224	*n* = 888	*n* = 181	*n* = 1475	*n* = 236	*n* = 835	*n* = 4932	*n* = 530	*n* = 560	*n* = 1817	*n* = 122
Crude	Mean	129.1	125.6	121.8	114.9	114	113.4	128.2	125.6	122.7	135.1	135.2	134.6
	*p* for trend	<0.0001			0.2341			<0.0001			0.8804		
Adjusted for age, sex	LS means	128.4	127.5	126.1	118.4	117.4	116.6	129.2	127.9	126.7	135.4	135.5	134.2
	*p* for trend	0.001			0.1034			0.0037			0.7621		
Adjusted for multivariate model	LS means	126.3	125.1	123.1	117.6	116.6	114.9	126.8	125.3	123.3	132.6	132.3	131.6
	*p* for trend	<0.0001			0.0045			0.0003			0.5952		

Men													
		Total *n* = 2831			20–39 years *n* = 449			40–64 years *n* = 1367			≥65 years *n* = 1015		
		<2500 g	2500–3499 g	≥3500 g	<2500 g	2500–3499 g	≥3500 g	<2500 g	2500–3499 g	≥3500 g	<2500 g	2500–3499 g	≥3500 g
		*n* = 454	*n* = 2131	*n* = 246	*n* = 42	*n* = 348	*n* = 59	*n* = 186	*n* = 1062	*n* = 119	*n* = 226	*n* = 721	*n* = 68
Crude	Mean	135.1	132.5	130.8	122.3	124.0	124.4	136.4	132.0	130.9	136.4	137.4	136.3
	*p* for trend	0.0003			0.4191			0.001			0.7059		
Adjusted for age	LS means	133.6	132.7	132.3	122.1	124.0	124.2	135.7	132.0	132.2	136.4	137.4	136.1
	*p* for trend	0.2372			0.4361			0.0203			0.7371		
Adjusted for multivariate model	LS means	132.9	131.4	130.3	122.4	123.8	124.1	136.2	131.3	129.7	135.2	136.2	135.4
	*p* for trend	0.0283			0.5647			0.0005			0.7562		

Women													
		Total *n* = 7857			20–39 years *n* = 1443			40–64 years *n* = 4930			≥65 years *n* = 1484		
		<2500 g	2500–3499 g	≥3500 g	<2500 g	2500–3499 g	≥3500 g	<2500 g	2500–3499 g	≥3500 g	<2500 g	2500–3499 g	≥3500 g
		*n* = 1122	*n* = 6093	*n* = 642	*n* = 139	*n* =1127	*n* = 177	*n* = 649	*n* = 3870	*n* = 411	*n* = 334	*n* = 1096	*n* = 54
Crude	Mean	126.7	123.2	118.4	112.6	110.9	109.7	125.9	123.8	120.3	134.3	133.7	132.5
	*p* for trend	<0.0001			0.0171			<0.0001			0.4811		
Adjusted for age	LS means	124.2	123.3	121.7	112.7	110.8	109.8	124.5	123.8	122.2	134.2	133.8	132.2
	*p* for trend	0.0025			0.0145			0.039			0.5247		
Adjusted for multivariate model	LS means	122.2	121.1	118.9	112.7	110.9	108.4	122.3	121.7	119.6	130.5	129.6	129.4
	*p* for trend	0.0002			0.0002			0.026			0.4027		

Multivariate model: adjusted for age, sex, BMI, total cholesterol, HbA1c, smoking status, and alcohol status

*LS* least square

### ORs of hypertension based on the combination of birth weight and current BMI

**Fig. 2:**
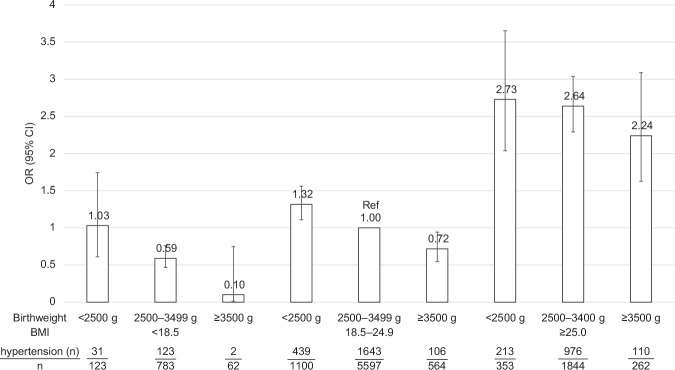
Adjusted ORs for having hypertension based on a combination of birth weight and current BMI. Hypertension was defined as SBP ≥ 140 mmHg and/or DBP ≥90 mmHg and/or under treatment. Adjusted ORs were calculated using age, sex, total cholesterol, HbA1c, smoking status, and alcohol status. CI confidence intervals, BMI body mass index, DBP diastolic blood pressure, OR odds ratio, Ref reference, SBP systolic blood pressure

Figure 2 and Table 3 show the ORs and 95% CI for having hypertension based on the combination of birth weight and current BMI. The prevalence of hypertension was the highest in the LBW and BMI ≥25.0 kg/m^2^ group, the ORs (95% CI) were 2.73 (2.04–3.65) compared to the reference group in the multivariate model. On the other hand, the lowest prevalence of hypertension was found in the birth weight ≥3500 g and BMI <18.5 kg/m^2^ group, the ORs (95% CI) were 0.10 (0.01–0.75). The risk in the LBW and BMI ≤ 18.5 kg/m^2^ group was not significantly different from the reference group (i.e., normal birth weight and low BMI).

**Table 3:** Adjusted ORs for having hypertension based on a combination of birth weight and current BMI

	Birth weight category			p for interaction
	<2500 g	2500–3499 g	≥3500 g	
Non obesity				
<18.5	1.03 (0.61–1.74)	0.59 (0.46–0.76)	0.10 (0.01–0.75)	
18.5–24.9	1.32 (1.11–1.56)	1.00 (Ref)	0.72 (0.55–0.94)	
				0.352
Obesity				
≥25.0	2.73 (2.04–3.65)	2.64 (2.29–3.04)	2.24 (1.63–3.09)	

Hypertension was defined as SBP ≥140 mmHg and/or DBP ≥90 mmHg and/or under treatment

Adjusted ORs were calculated using age, sex, total cholesterol, HbA1c, smoking status, and alcohol status

*BMI* body mass index, *DBP* diastolic blood pressure, *Ref* reference, *SBP* systolic blood pressure, *SD* standard deviation

In terms of BMI, the risk of hypertension was consistently higher among obese participants irrespective of their birth weight, while the risk of hypertension was the highest in the LBW group in BMI ≥25.0 kg/m^2^.

There was no significant interaction between birth weight category and obesity in interaction term analysis. We performed an additional analysis limited <65 years, these associations remained the same (Supplemental Table 1, Table 2, and Fig. 1).

## Discussion

In this study, we clarified that LBW was associated with subsequent hypertension irrespective of current BMI and showed that even participants who had a lower weight might prevent hypertension via weight control. This is the first study to examine the risk of hypertension associated with the combination of birth weight and current BMI. The large sample size allowed us to analyze the combined association of birth weight and current BMI.

We found that LBW was associated with the subsequent risk of hypertension. There was a significant inverse association between birth weight and SBP in younger and middle-aged adults. Furthermore, birth weight and BMI were independently associated with hypertension. Participants in the LBW and current obesity subgroups had the highest risk of hypertension.

In 1986, Barker et al. published the first series of cohort studies suggesting that cardiovascular disease was inversely associated with birth weight [16]. Epidemiological studies from several countries have reported LBW to be associated with an increased risk of lifestyle diseases such as hypertension, diabetes, and hyperlipidemia later in life [4, 5]. Similar studies have been reported in Japan [17–19]. A meta-analysis by Knop et al. suggested that birth weight was inversely associated with the risk of hypertension in adulthood [4].

In previous studies, an inverse relationship between birth weight and BP was reported at different ages, that is, childhood [11], adolescence [20], younger age [11, 12], middle age [11, 12, 21], and old age [11, 22, 23]. In our subgroup analysis, there was an inverse association between birth weight and SBP in those aged <65 years, but no association was observed in those aged ≥65 years.

We considered several possibilities regarding the lack of association between LBW and subsequent hypertension in the older age groups. One possible reason for the lack of association in our study among those 65 aged and older may be that previous studies used birth weight records, whereas this study used the recall of participants. From 1942, the Handbook of Pregnant Mothers has been used in Japan [24]. Furthermore, from 1948, the Maternal and Child Health Handbook has been widely used in Japan [24]. This handbook contains records on pregnancy, delivery, child development, and healthcare. Thus, most participants aged under 65 years might have obtained sufficient birth weight; conversely, those over the age of 65 did not. Although the Handbook of Pregnant Mothers was distributed to pregnant women from 1942, therefore, those 65–70 years had a lower chance of accessing their recorded birth weights. This might explain the age difference between the participants who reported their birth weight and those who did not. We also considered that uncertainty in birthweight recall among older participants, even when they reported their birth weight, may have obscured these associations. Another possibility is that lifestyle habits, such as exercise, diet, and sleeping, have a stronger influence on BP than birth weight in old age.

Obesity has traditionally been a risk factor for hypertension, and the results in Fig. 2 confirm that obesity was a risk factor for hypertension in our study. In the BMI ≥25.0 kg/m^2^ group, all birth weight subgroups had an increased risk of hypertension, especially the LBW group. In the BMI < 25.0 kg/m^2^ groups, the LBW group had a higher risk of hypertension than in the birth weight 2500–3499 g and BMI 18.5–24.9 kg/m^2^ group, and the birth weight ≥3500 g kg/m^2^ group had a lower risk of hypertension. In our study, the highest risk of hypertension was observed in the participants with LBW and obesity. These findings are consistent with those of previous studies.

Previous studies examining the association between birth weight and obesity in hypertension have reported similar results from childhood to adulthood [11, 23, 25]. A reduced number of nephrons have been suggested to play an important role in the mechanism of hypertension in LBW [26–29]. Approximately 60% of nephrons develop during the third trimester of pregnancy up to 36 weeks, and no new nephrons are formed after birth [30]. LBW infants with growth restriction or preterm delivery have fewer nephrons [30, 31]. Nephrogenesis occurs during preterm birth and postnatal nephrogenesis continues after preterm birth for 40 days [30, 31]. However, morphologically abnormal glomeruli were present in the kidneys of preterm infants [30, 31]. Similarly, renal development during fetal growth restriction (FGR) results in a reduced number of nephrons and morphologically abnormal glomeruli [31]. The reduction in the number of normal nephrons leads to the overloading of each nephron.

Obesity is also associated with hypertension. Renal salt retention is a major mechanism underlying obesity-associated hypertension. Obesity increases glomerular filtration due to the activation of renal sympathetic activity, renin-angiotensin-aldosterone system, and insulin resistance [32]. The imbalance between structurally limited filtration in LBW and the increased load on the kidney in obesity tends to increase BP to maintain sodium homeostasis [33].

Individuals born with LBW already have a potential risk for hypertension compared to those born with normal birth weight having normal BMI in adulthood. However, prevention of obesity may reduce this risk. Our results showed that when individuals with LBW maintained a BMI <18.5 kg/m^2^, they had a reduced risk of hypertension compared to those with normal birth weight and BMI. Understanding the risk factors of lifestyle-related diseases, including obesity, and implementing preferable lifestyle habits from an early age at the parents’ initiative would make it possible to reduce the potential risk factors and prevent the development of hypertension in adulthood in this group. On the other hand, in our study, those with a high birth weight had a lower prevalence of hypertension in adulthood, but the risk of hypertension increased with obesity, and maintaining a normal BMI range was important even for the high birth weight group.

The strength of this study is that the recalled birth weight was reliable, especially for subjects born after 1942, that is <65 years. A previous study showed high agreement between self-reported birth weight and recorded birth weight. Therefore, self-reported birth weight is sufficiently reliable for use in epidemiological studies [34].

A limitation of this study was the lack of information on gestational age. We were unable to distinguish between premature and growth-restricted infants. At the same birth weight, preterm and fetal growth-restricted infants differ in the degree of immature organ maturation according to the gestational period [35, 36].

### Perspective of Asia

In Asia, there is a high proportion of people with low birth weight [7]. On the other hand, there is also a high proportion of those with low BMI [37]. This study showed that people born with low birth weight may be able to prevent the onset of hypertension by controlling their later weight appropriately. We believe that our study might contribute better BP control, especially among women with lower birth weight. Our report is the first finding in Asia, replication studies in other populations are expected.

## Conclusions

There was a significant association between LBW and hypertension in the Japanese population. Participants with LBW had a higher risk of hypertension than those with higher birth weight. Furthermore, LBW and obesity were associated with a higher risk of hypertension than normal or high birth weight groups with normal BMI. However, even in participants with a lower birth weight, the risk of hypertension could be reduced when they maintained an optimal BMI. Therefore, maintaining normal weight may be effective in preventing hypertension, especially in individuals with LBW.

## Supplementary information

Supplemental Table 1 and 2

Supplemental figure

Supplemental Figure 1
