# Supplementary material for: The association of birth weight and current BMI on the risk of hypertension: the Tohoku medical megabank community-based cohort study
Source: Hypertens Res. 2024 Aug 8;47(11):3025–34. doi: 10.1038/s41440-024-01827-z (PMC11534687; doi:10.1038/s41440-024-01827-z)
Supplement: Supplementary file 1 — Supplemental Table 1 and 2 [file 41440_2024_1827_MOESM1_ESM.docx]

**Supplemental Table 1.** Birth weight and least square means of systolic blood pressure (limited <65 years old)

|  |  | Total　n=8,189 | | | Men　n=1,816 | | | Women　n=6,373 | | |
| --- | --- | --- | --- | --- | --- | --- | --- | --- | --- | --- |
|  |  | <2500 g | 2500–3499 g | ≥3500 g | <2500 g | 2500–3499 g | ≥3500 g | <2500 g | 2500–3499 g | ≥3500 g |
|  |  | n=1,016 | n=6,407 | n=766 | n=228 | n=1,410 | n=178 | n=788 | n=4,997 | n=588 |
| Crude | Mean | 125.8 | 122.9 | 119.8 | 133.8 | 130.0 | 128.8 | 123.5 | 120.9 | 117.1 |
|  | p for trend | <0.0001 |  |  | 0.0006 |  |  | <0.0001 |  |  |
| Adjusted for age, sex | LS means | 126.9 | 125.5 | 124.3 | 132.7 | 130.0 | 130.2 | 121.9 | 120.9 | 119.3 |
|  | p for trend | 0.0003 |  |  | 0.0637 |  |  | 0.0016 |  |  |
| Adjusted for multivariate model | LS means | 124.8 | 123.2 | 121.1 | 132.7 | 129.3 | 128.2 | 120.1 | 119.1 | 116.7 |
|  | p for trend | <0.0001 |  |  | 0.0026 |  |  | 0.0002 |  |  |

Multivariate model: adjusted for age, sex, BMI, total cholesterol, HbA1c, smoking status, and alcohol status

LS, least square.

**Supplemental Table 2.** Adjusted ORs for having hypertension based on the combination of birth weight and current BMI (limited <65 years old)

|  | Birth weight category | | |  |
| --- | --- | --- | --- | --- |
|  | <2500 g | 2500–3499 g | ≥3500 g | p for interaction |
| Non obesity |  |  |  |  |
| <18.5 | 1.02 (0.53–1.96) | 0.57 (0.43–0.77) |  |  |
| 18.5–24.9 | 1.33 (1.07–1.65) | 1.00 (Ref) | 0.76 (0.56–1.03) |  |
|  |  |  |  | 0.736 |
| Obesity |  |  |  |  |
| ≥25.0 | 3.02 (2.15–4.25) | 2.68 (2.28–3.16) | 2.43 (1.72–3.43) |  |

Hypertension was defined as SBP ≥140 mmHg and/or DBP ≥90 mmHg and/or under treatment.

CI, confidence interval; DBP, diastolic blood pressure; LS, least square; OR, odds ratio; Ref, reference; SBP, systolic blood pressure.
