# Supplementary figures and images for: The association of birth weight and current BMI on the risk of hypertension: the Tohoku medical megabank community-based cohort study
Source: Hypertens Res. 2024 Aug 8;47(11):3025–34. doi: 10.1038/s41440-024-01827-z (PMC11534687; doi:10.1038/s41440-024-01827-z)

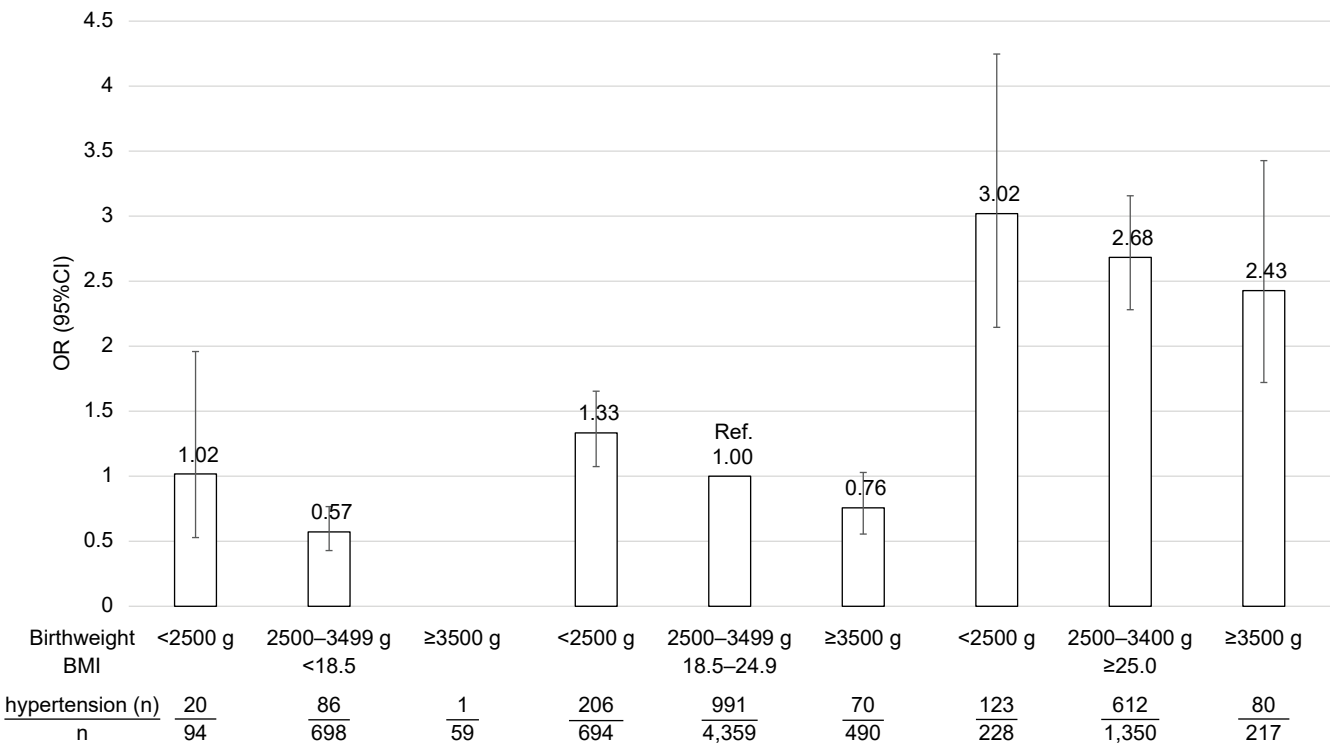

Supplement: Supplementary file 2 — Supplemental figure [file 41440_2024_1827_MOESM2_ESM.pdf]
