# Supplementary material for: The association of birth weight and current BMI on the risk of hypertension: the Tohoku medical megabank community-based cohort study
Source: Hypertens Res. 2024 Aug 8;47(11):3025–34. doi: 10.1038/s41440-024-01827-z (PMC11534687; doi:10.1038/s41440-024-01827-z)
Supplement: Supplementary file 3 — Supplemental Figure 1 [file 41440_2024_1827_MOESM3_ESM.docx]

**Supplemental Figure 1.** Adjusted ORs for having hypertension based on a combination of birth weight and current BMI (limited <65 years old).

Hypertension was defined as SBP ≥140 mmHg and/or DBP ≥90 mmHg and/or under treatment.

Adjusted ORs were calculated using age, sex, total cholesterol, HbA1c, smoking status, and alcohol status.

CI, confidence intervals; BMI, body mass index; DBP, diastolic blood pressure; OR, odds ratio; Ref, reference; SBP, systolic blood pressure.
